# Suggested Integral Analysis for Chaos-Based Image Cryptosystems

**DOI:** 10.3390/e21080815

**Published:** 2019-08-20

**Authors:** Miguel Angel Murillo-Escobar, Manuel Omar Meranza-Castillón, Rosa Martha López-Gutiérrez, César Cruz-Hernández

**Affiliations:** 1 Electronics and Telecommunication Department, Scientific Research and Advanced Studies Center of Ensenada (CICESE), Ensenada BC 22860, Mexico; 2 Engineering, Architecture and Design Faculty, Autonomous University of Baja California (UABC), Ensenada BC 22860, Mexico

**Keywords:** chaotic cryptography, security analysis, image information entropy

## Abstract

Currently, chaos-based cryptosystems are being proposed in the literature to provide confidentiality for digital images, since the diffusion effect in the Advance Encryption Standard (AES) algorithm is weak. Security is the most important challenge to assess in cryptosystems according to the National Institute of Standard and Technology (NIST), then cost and performance, and finally algorithm and implementation. Recent chaos-based image encryption algorithms present basic security analysis, which could make them insecure for some applications. In this paper, we suggest an integral analysis framework related to comprehensive security analysis, cost and performance, and the algorithm and implementation for chaos-based image cryptosystems. The proposed guideline based on 20 analysis points can assist new cryptographic designers to present an integral analysis of new algorithms. Future comparisons of new schemes can be more consistent in terms of security and efficiency. In addition, we present aspects regarding digital chaos implementation, chaos validation, and key definition to improve the security of the overall cryptosystem. The suggested guideline does not guarantee security, and it does not intend to limit the liberty to implement new analysis. However, it provides for the first time in the literature a solid basis about integral analysis for chaos-based image cryptosystems as an effective approach to improve security.

## 1. Introduction

Information security in modern digital systems and telecommunication networks has been one of the major concerns during the last few decades with an increasing research area in cryptography to provide confidentiality and protect secrets from eavesdroppers, intruders, adversaries, or enemies by using cryptosystems. Transmitting or storing confidential images over insecure channels can have a high risk. Thus, such information must be protected prior to sending or storing, e.g., clinical images or radiological photos (for diagnosis or consultation) in telemedicine, personal identifiers such face, iris, or fingerprint images (for control access systems) in biometric systems, or satellite maps in military are some potential applications for image encryption. Basically, an encryption algorithm will map the plain text (recognizable message) to encrypted text (unrecognizable message) with some specific key, and a decryption algorithm will map the encrypted text to plain text with the corresponding specific key. Cryptosystems can be performed by hand methods, machine methods, or software based on Shannon’s model. In cryptanalysis, several kinds of attacks can be implemented to break a cryptosystem such as side-channel attack, physical attack, invasive attack, radio frequency attack, impersonation attack, chosen/known plain text attack, differential attack, exhaustive search attack, among others. For example, a physical attack in cryptographic implementations includes all kind of attacks related to physical means against encryption devices, e.g., a microcontroller-based cryptosystem or a smart card [1].

In modern cryptography, asymmetric-key and symmetric-key are the most common methods of conventional cryptosystems. Asymmetric-key schemes use a pair of keys, i.e., private key and public key, such as Rivest, Shamir, and Adleman (RSA), which is an encryption algorithm for data confidentiality with a common key length between 1024 and 2048 bits [2]; or Elliptic-curve Diffie–Hellman (ECDH), which is an anonymous key agreement protocol to establish a shared secret key over an insecure channel that can be used in a symmetric-key encryption scheme [3]. These kinds of methods are based on intractable mathematical problems, i.e., problems that can be resolved in theory, but in practice take too long for their solution (e.g., factoring a number into primes, integer factorization, or discrete logarithm). In symmetric-key cryptosystems, the same key is used for encryption and decryption, which is considered secret, and it must be shared between parties securely. In addition, there are block ciphers and stream ciphers. Block cipher encrypts plain data in fixed-length groups of bits such as the Advanced Encryption Standard (AES), the Triple Data Encryption Standard (3DES), or the International Data Encryption Algorithm (IDEA). Stream cipher encrypts plain data bit-by-bit with a pseudorandom keystream such as Rivest Cipher 4 (RC4), Rabbit, HC-128/256, Salsa 20/12, and Trivium, among others. These schemes are based on an iterated process or a series of linked mathematical operations, i.e., the Feistel network and the substitution-permutation network (SPN), where several rounds of substitution boxes (S-boxes) and permutation boxes (P-boxes) are implemented with exclusive-or (XOR) or bitwise rotation. In addition, key lengths of 128, 192, or 256 bits are used [4]. According to the National Institute of Standard and Technology (NIST), a key of 15360-bit in the RSA algorithm is equivalent in strength to a 256-bit one in the AES algorithm [5]. Although asymmetric-key schemes use two keys to increase the security of the cryptosystem, symmetric-key schemes are typically faster and less expensive to implement. In this sense, symmetric-key cryptography is typically used for data encryption, and asymmetric-key cryptography is used for digital signatures or secure-key exchange protocols. Because the diffusion effect in the AES algorithm is weak, conventional symmetric-key cryptosystems as AES are not suitable to encrypt digital images securely and efficiently.

In the last years of the Twenty Century, the scientific community found an interesting research area related to chaos-based cryptography (non-conventional cryptography), since chaotic systems present properties such as extreme sensibility to initial conditions and control parameters, ergodicity, mixing data, non-linearity, random-like behavior, aperiodic dynamics, and determinism, which are strongly related to cryptographic properties such as complexity in the source system, pseudorandomness, and an excellent substitution-permutation process to provide security and efficiency in chaos-based image cryptosystems. Analog and digital chaos-based cryptosystems have been proposed in the literature. The analog implementation was first proposed in the 1990s, which is based on chaotic synchronization techniques first proposed by Pecora and Carroll in 1990 [6] and analog circuits (hardware-based key) to transmit analogical or digital information (such as digital images) by using techniques such as chaos modulation and chaos masking proposed by Oppenheim et al. [7], or chaos spreading spectrum proposed by Cuomo et al. [8]. Based on such techniques, analog chaos-based cryptosystems have been proposed in the literature; see, e.g., [9,10,11,12].

On the other hand, Fridrich proposed the permutation-diffusion architecture for digital chaos-based image encryption in 1998 [13]. Based on Fridrich’s scheme, digital chaos-based cryptosystems or hyperchaos-based cryptosystems have been proposed based on computer simulation and the symmetric-key method, e.g., gray-scale image encryption [14,15,16], RGB color image encryption [17,18,19,20], H.264 video encryption [21], text encryption [22], biometric encryption [23], and biosignal encryption [24].

Recent chaos-based image cryptosystems (2015–2019) present “basic” security analysis (i.e., key space, key sensitivity, plain text sensitivity, graphic histogram, graphic correlation, correlation coefficient, and information entropy) or partial security analysis and speed performance (for encryption/decryption); see, e.g., [25,26,27,28,29,30,31,32,33,34,35,36,37,38,39,40,41,42,43]. Table 1 summarizes the security analysis of recent chaos-based image cryptosystems. NIST considers security as the most important criteria in a cryptographic evaluation, following the cost, algorithm, and finally, implementation [44,45]. Recent chaos-based image cryptosystems or hyperchaos-based cryptosystems do not present a comprehensive security analysis according to Table 1, which could make them unreliable and insecure for some applications (e.g., telemedicine, biometrics systems, or in military affairs). Even analysis about cost end performance and the algorithm and implementation are not considered in detail. Such differences in security terms between recent chaos-based algorithms are due to the lack of frameworks or guidelines for security analysis, which is the main motivation of this paper.

Security analysis is a testing technique to determine if a cryptosystem protects the information adequately, and it must include analysis against all known attacks efficiently, since excellent performance and implementation advantages of a broken cryptosystem are irrelevant. Therefore, every chaos-based cryptosystem must provide enough information regarding their security and efficiency.

Few efforts to establish the cryptographic requirements for chaos-based cryptography have been proposed in the literature. In 2006, Álvarez and Li proposed some basic cryptographic requirements for chaos-based cryptosystems with the aim of assisting designers to present their work in a more systematic and rigorous way; several recommendations based on 17 suggested rules (related to implementation, key management, and security analysis) were proposed for analog chaos-based secure communications [46]. In 2018, Özkaynak proposed a roadmap for the security analysis of new chaos-based image cryptosystems, since many proposals that only used the unified averaged changed intensity (UACI) and the number of changing pixel rate (NPCR) tests to evaluate security with respect to differential attacks have been observed to be weak; in addition, a checklist and design guide were proposed to make more robust analyses, which is based on 12 steps [47]. The checklist of Özkaynak has been implemented in recent chaos-based image cryptosystem to complement the discussion of the proposed scheme in [40]. In the same year, Preishuber et al. showed that computational effort and security benefits are highly questionable for chaos-based image cryptosystems in comparison with conventional algorithms; even they demonstrated that statistical tests used to assess the security of chaos-based encryption schemes are insufficient metrics for security analysis. They pointed out that further methodologies for the security assessment for chaos-based encryption schemes need to be entirely reconsidered [48].

In this paper, we present a suggested integral analysis framework based on a comprehensive security analysis, cost and performance analysis, and algorithm and implementation details, with the aim to establish a basis in security analysis for chaos-based image cryptosystems. The proposed guideline based on 20 analysis points can assist cryptographic designers to present a common study about an integral security analysis of their new cryptosystems with the aim to show exhaustive information about security, reliability, integrity, protection of secrets, easy of use, speed, and acceptable costs. If the authors consider this guideline, future comparisons of new proposals can be more consistent in terms of security and efficiency, which can help to develop more secure and efficient encryption algorithms. The suggested framework is based on a literature review, new analysis (such as histogram uniformity, graphic autocorrelation, and floating frequency), and the evaluation criteria of NIST for cryptosystems (security, cost and performance, and the algorithm and implementation). We use the chaos-based RGB image encryption algorithm [25] to determine the results of the suggested framework. Furthermore, we present aspects regarding digital chaos implementation, chaos validation, and key definition with the aim to improve the overall security of new cryptosystems. Designers are invited to follow the recommendations of this work, but without limiting their liberty, new analysis, and their own creativity to establish the security of their new cryptosystems.

The rest of the paper is organized as follows. In Section 2, digital chaos aspects are presented to improve the overall cryptosystem security. The suggested framework related to a comprehensive security analysis is given in Section 3. The cost and performance analysis are shown in Section 4. In Section 5, the algorithm and implementation details of chaos-based digital cryptosystems are presented. Finally, the conclusions are mentioned in Section 6.

## 2. Digital Chaos

Cryptosystems based on digital chaos have attracted more attention than its analog version, since discrete chaotic systems or chaotic maps present advantages related to security, performance, flexibility, and cost. In 2003, Li proposed some basic suggestions to design fast and low-cost digital chaotic ciphers: choosing the simplest chaotic map to achieve better performance, using the minimum iterations in chaotic maps, avoiding use of multiple chaotic maps in software implementations, using fixed-point arithmetic as much as possible, avoiding chaotic maps with floating-point arithmetic requirements, and adopting a parallel mechanism in hardware implementations [49].

Digital chaos implementation implies finite numbers either with floating-point arithmetic, fixed-point arithmetic, or other arithmetic at a reasonable digital word size. Chaos degradation occurs principally for continuous chaotic systems, when a numerical solution such as Euler or Runge–Kutta (RK4) is applied. On the other hand, chaotic maps are discrete by nature. Nevertheless, a chaotic sequence can fall into a short chaotic cycle when it is realized in a low digital word size. It is known that fixed-point arithmetic requires low-power, low-cost, simpler, and smaller hardware than floating-point arithmetic for a reasonable word size in signal processing. However, the floating-point has a larger dynamic range and can represent numbers of the real world. Therefore, the floating-point reduces the risk of overflow, quantization errors, and the need for scaling. For example, when a chaos-based cryptosystem is implemented in MATLAB, the format long is used for a 64-bit word size in fixed-point arithmetic, whereas format long E is for a 64-bit word size in floating-point.

In the last two decades, designers have been implementing cryptosystems based on one-dimensional chaotic maps such as the logistic map or piecewise linear chaotic maps and with two-dimensional chaotic maps such as the 2D logistic map, Hénon map, Arnold’s cat map, the Duffing map, the Ikeda map, the standard map, the Tinkerbell map, and the Baker map.

### 2.1. Chaos Validation

The sequences used for the encryption process must be validated chaotically to produce cryptograms with good statistical properties. The initial conditions and control parameter of chaotic maps are considered as the secret key. Therefore, control parameters must be defined adequately to avoid non-chaotic regimes. For example, the logistic map produces chaos when the control parameter is between 3.57 and four. Nevertheless, there are some values where the dynamics become periodic. A robust numerical validation of the chaotic sequence must be presented such as the Lyapunov exponent [50] or the recent 0–1 Gottwald–Melbourne test [51]. A positive Lyapunov exponent indicates chaos, and a value close to one in the Gottwald–Melbourne test indicates chaos.

In some cases, the control parameter can be adjusted to guarantee chaos. For example, if the control parameter of the logistic map starts with 3.9 and a double precision (64 bits) floating-point format is adopted, then we can manipulate 14 digits (but reducing the complexity from $2^{52}$ to $2^{49}$, approximately), but it produces always chaotic sequences (strong keys). We suggest to complement this with some numerical validation by using several tests with several secret keys selected randomly to provide robustness.

### 2.2. Key Definition

Recent chaos-based digital cryptosystems reported in the literature are based on fixed-point or floating-point arithmetic with double precision. Therefore, 15 decimal digits of initial conditions and control parameters are defined as the secret key, which make it complicated to implement in real applications. For example, Niyat et al. in 2017 [33] defined the secret key with 135 decimal digits based on six initial conditions and three control parameters. In this sense, there are recent cryptosystems (see, e.g., [27,28,30,32]). In some cryptosystems, the secret key is defined with a stream of hexadecimal numbers, which can be more practical for implementation and sharing between parties. The initial conditions and control parameters are determined from this secret key by using some mathematical process; see, e.g., [25,31,52]. We suggest including some efficient algorithm to implement practical keys with n>128 bits such as used in conventional cryptography. For example, the one-dimensional logistic map is described as follows:

(1) $$x_{i+1}=ax_{i}\left(1-x_{i}\right),$$

where $x_{i}\in \left(0,1\right)$ is the discrete state with initial condition $x_{0}\in \left(0,1\right)$ and control parameter a∈(3.57,4) to produce chaotic sequences. If this chaotic map is implemented by using IEEE 754 double floating-point arithmetic (until 15 decimals) and all decimals are considered, the maximum complexity for state *x* is $2^{52}$ and for control parameter *a* is $2^{52}$. A practical key could be the hexadecimal format such as used in conventional cryptography. In this case, we can define a secret key *H* with 26 hexadecimal digits, and an algorithm determines the value for *x* and *a* of the logistic map. An example of this process can be defined as follows:

- Calculate constants $A=\frac{{\left(H_{1},H_{2},\ldots ,H_{13}\right)}_{10}}{2^{52}}$ and $B=\frac{{\left(H_{14},H_{15},\ldots ,H_{26}\right)}_{10}}{2^{52}}$.
- Assign constants to the initial condition and control parameter with $x_{0}=A$ and a=3.9+(B∗0.1).

The key space and key sensitivity must be reviewed carefully when this kind of process is included in a cryptosystem (see Section 3.1).

### 2.3. Encryption Algorithm

The substitution-permutation network has been adopted in most chaos-based encryption algorithms proposed in the literature, with one or several rounds of each process to increase security. The main idea is to change the value of each plain element and change its position according to chaotic sequences to produce cryptograms that are statistically efficient.

On the other hand, cryptanalysis has been applied in chaos-based image cryptosystems by using the powerful chose/known plain image attacks [53,54,55]. The chaotic keystream for encryption could be derived from both the secret key and the plain image to provide robustness against this kind of attack. Nevertheless, if an error occurs in the transmitted cryptogram, than it could be impossible to decrypt such a cryptogram. Some recent chaos-based digital cryptosystems do not considered this important fact. In addition, it provides higher sensitivity against plain images. Recently, some advances over the algorithm design have been addressed to resist the chosen/known plain image attack, where the plain image is included for the chaotic keystream used in the encryption process [25,36], adding random values to the plain image before encryption [52], or by using the SHA256 algorithm (hash function) for the plain image [16,56], but the receiver must have the same hash value for each different image encryption; it could be included in the cryptogram, but if it is damaged during transmission, the receiver cannot retrieve the original image; or the sender could securely exchange it with the receiver each time a different plain image is encrypted.

## 3. Security Analysis

In this section, we present several statistical security analyses that can be applied over chaos-based image cryptosystems. The presented security analysis is based on a literature review, and we propose three new statistical analyses such as histogram uniformity, graphic autocorrelation, and floating frequency. Table 1 shows us that most authors included basic tests such as key size, sensitivity of the secret key and plain image, information entropy, histograms, and correlation. In some cases, other analyses are considered such as encryption quality, noise, and occlusion. Such differences make it difficult for a robust comparison among cryptosystems in terms of security or efficiency.

Consider an RGB color plain image P∈(0,255) of M×N×3 pixels, where *M* is the rows, *N* is the columns, and three is the color component with dimension M×N for red, green, and blue. A gray-scale plain image P∈(0,255) of M×N pixels has a bit depth of eight bits, whereas the color image has 24 bits (eight bits for each component). Therefore, a color plain image of 256×256 has a bulk data of 196.608 KB (which can vary according to the image format). The results of the proposed framework are based on the chaos-based RBG encryption algorithm proposed in [25].

**Figure 1:**
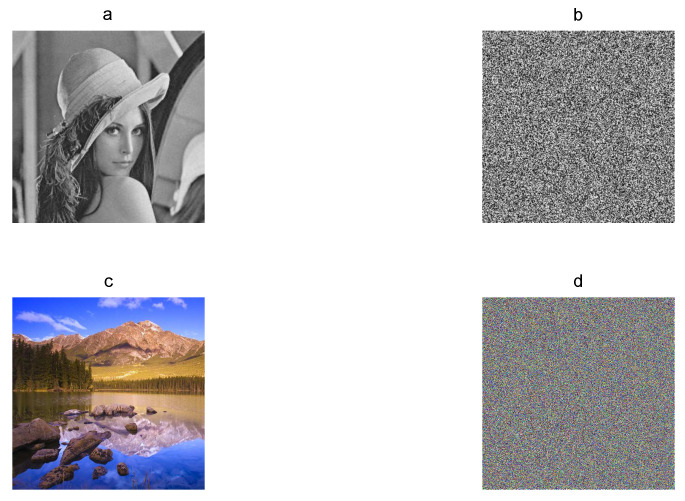
Plain images and their corresponding encrypted images: (**a**) Lena plain image, (**b**) Lena encrypted image, (**c**) Landscape plain image, and (**d**) Landscape encrypted image.

First at all, a chaos-based image encryption algorithm must produce encrypted image *E* visually without clear information. In Figure 1, the gray-scaleLena plain image of 256×256 pixels and the RGB color Landscape plain image of 512×512 pixels are shown with their corresponding encrypted images, which do not present clear information for human eyes.

### 3.1. Point 1: Key Space

The key is defined as a bit string that determines how the algorithm will map the plain image *P* to encrypted image *E*. It is considered highly important from a security point of view, since all about the cryptosystem is know by the adversary except the key (Kerckhoffs’ principle). The most obvious attack over the cryptosystem is the exhaustive search attack or brute-force attack, where each possible key is used until the cryptosystem is broken. In addition, adversaries have access to latest technology including supercomputers. The key space is designed to be large enough to resit this kind of attack considering the actual technology, e.g., the fastest supercomputer today (Summit) is capable of 200 PFLOPS ($10^{15}$ floating-point operation per second) or 200,000 trillion calculations per second. The key space must be more than 100 bits according to Álvarez and Li [46]. This key size requires $1.99\times 10^{23}$ years (Equation (2)) to break a cryptosystem with the above supercomputer and assuming 1000 FLOPS per checking,

(2) $$Years=\frac{Key\;Combinations\times 1000}{FLOPS}\times 31536000$$

Most of the chaos-based digital cryptosystems implemented in MATLAB use the IEEE Standard for Floating-Point Arithmetic (IEEE 754) for floating-point computation in 64 bits (double precision) to represent real numbers of chaotic states with the aim of avoiding digital degradation and providing a huge key space. Note that not always all 64 bits (1 bit for the sign, 11 bits for the exponent, and 52 bits for the significant precision) are used for each variable of chaotic maps (i.e., chaotic states or control parameters). Therefore, the cryptographic designer must take care about the range of these values to estimate the key space. For example, the well-known 2D Hénon map is defined by:

(3) $$\begin{array}{c} x_{i+1}=1-bx_{i}^{2}+y_{i},\\ y_{i+1}=cx_{i}, \end{array}$$

where *b* and *c* are the two control parameters and $x_{0}$ and $y_{0}$ are the two initial conditions. The dynamics of the map in Equation (3) is chaotic when b=1.4, c=0.3, $x_{0}=0$, and $y_{0}=0$. The sign and integer section of the IEEE 754 format (12 bits) cannot be considered as part of the key space, since it does not vary enough in this case. Nevertheless, the decimal part (significant precision) can vary with thousands of possibilities. If control parameters start with b=1.4XXXXXXXXXXXXXX and c=0.3XXXXXXXXXXXXXX, then *b* and *c* will have a complexity of $2^{48}$ (approximate) instead of $2^{52}$, since just 14 of the 15 decimals can change their value. Therefore, the key space by using the Hénon map (Equation (3)) in this case is about $2^{52+52+48+48}=2^{200}$, i.e., a key of 200 bits. These bits can be divided by four to have 50 hexadecimal numbers, and an effective algebraic process must be developed to determine all initial conditions and control parameters of the chaotic map.

In the encryption design, all the key space must produce chaotic behavior (i.e., strong keys) and avoid using weak keys (i.e., some secret key that produces non-chaotic dynamics). In some chaotic maps, the bifurcation diagram presents periodic windows combined with chaotic dynamics. In this sense, designers must avoid such values that generate periodic behavior. One method to determine if all the key spaces are strong is that the bifurcation diagram for each control parameter could be presented or calculating the Lyapunov exponent to validate chaos.

### 3.2. Point 2: Plain Image Sensitivity

In this subsection, the plain image sensitivity is determined by means of the next process. Two similar plain images $P_{1}$ and $P_{2}$ are encrypted by using the same secret key *K* to determine the difference between the resulting cryptograms $E_{1}$ and $E_{2}$. The plain image sensitivity level is based on two tests: NPCR (net pixel change rate) and UACI (unified average changing intensity). The NPCR determines how many pixels are different between $E_{1}$ and $E_{2}$ (in percentage), whereas the UACI determines the intensity difference average. The NPCR and UACI of an eight-bit gray-scale image of N×M pixels are calculated using Equation (4) and Equation (6), respectively.

(4) $$NPCR=\frac{100}{M\times N}\sum_{i=1}^{M}\sum_{j=1}^{N}W\left(i,j\right),$$

where

(5) $$W\left(i,j\right)=\left\{\begin{array}{cc} 0 & \mathrm{if}E_{1}\left(i,j\right)=E_{2}\left(i,j\right)\\ 1 & \mathrm{if}E_{1}\left(i,j\right)\neq E_{2}\left(i,j\right) \end{array}\right.$$

and:

(6) $$UACI=\frac{100}{M\times N\times 255}\sum_{i=1}^{M}\sum_{j=1}^{N}\left|E_{1}\left(i,j\right)-E_{2}\left(i,j\right)\right|,$$

where $E_{1}\left(i,j\right)$ and $E_{2}\left(i,j\right)$ are the pixel values of each encrypted image. NPCR and UACI are based on percentage to show the effect of small changes in the plain image. These metrics can be used as statistics to determine plain image sensitivity and resistance against differential attacks. In 2012, Wu et al. found the expected values of NPCR and UACI for ideally-encrypted images [57]. The critical value of NPCR for 256×256 image size is 99.5341% and 99.5717% for a 512×512 image size; thus, NPCR must be higher than these NPCR values. In UACI, there is an acceptance interval, which is 33.1594%–33.7677% for a 256×256 image size and 33.3115%–33.6156% for a 512×512 image size. In 2016, Belazi et al. proposed the expected NPCR and UACI values for a strong encryption scheme of an eight-bit image gray-scale, which is 99.6094% and 33.4635%, respectively [26]. Therefore, chaos-based image cryptosystems must present the above values for NPCR and UACI to validate high plain image sensitivity.

These quantities have been used to determine the robustness against differential analysis of several chaos-based image cryptography systems, but recent research points out that such quantities are not enough to be used as a security tool for differential attacks. Özkaynak in [58] proposed a comprehensive security road map to complement this problem, which is recommended to be considered for chaos-based image encryption designs.

### 3.3. Point 3: Graphic Histogram

**Figure 2:**
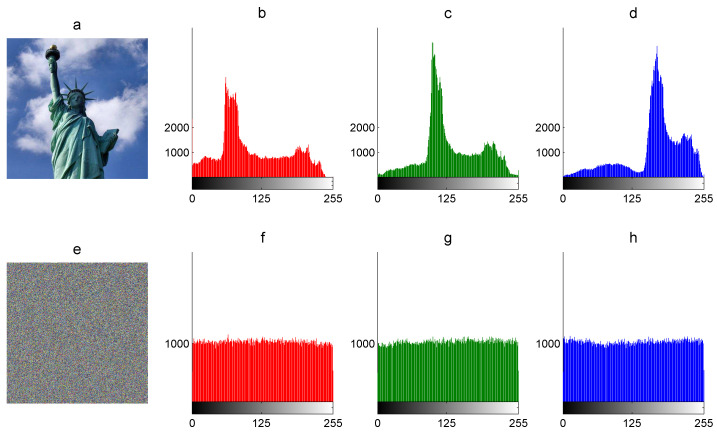
Graphic histograms: (**a**) color plain image *P*, (**b**) R-component of *P*, (**c**) G-component of *P*, (**d**) B-component of *P*, (**e**) encrypted image *E*, (**f**) R-component of *E*, (**g**) G-component of *E*, and (**h**) B-component of *E*.

The graphic histogram of an image is the visual inspection of statistical data and the tone of the image. It shows the frequency of pixel intensity values of the image graphically. The horizontal axis of the graphic represents the intensity variations, whereas the vertical axis represents the frequency of some particular intensity. The graphic histogram of the encrypted image must present similar frequencies for each pixel intensity value (uniform distribution) to avoid the leaking of statistical information related to the plain image and resist statistical attacks. For example, Figure 2 shows the graphic histograms for each R, B, and G component of The Statue of Liberty 512×512 plain image *P* and the graphic histograms of the corresponding encrypted image *E*.

### 3.4. Point 4: Histogram Statistics

The variance and standard deviation are metrics of dispersion implemented to support the results of visual inspection in graphic histograms. They measure how much the elements of a set of data vary with respect to each other around the mean. Two datasets may have the same average value (mean), but the variations can be drastically different.

The variance calculates the average difference between each of the values with respect to their central point (mean $\bar{x}$). This average is calculated by squaring each of the differences and calculating its mean again. The squaring process is used to eliminate the negative signs and to increase the variance of dispersed (non-uniform) datasets. On the other hand, the more uniform is the graphic histogram, the lower is the histogram variance, which is determined with the following expression:

(7) $$\alpha =\frac{1}{256}\sum_{i=1}^{256}{\left(x_{i}-\bar{x}\right)}^{2},$$

where:

(8) $$\bar{x}=\frac{M\times N}{256},$$

*x* is the frequency for each intensity value from 0–255 of the histogram, α is the histogram variance, and $\bar{x}$ is the mean of the histogram, i.e., it will be 256 for a 256×256 image size or 1024 for a 512×512 image size.

The standard deviation allow us to know the arithmetic average of fluctuations of the dataset with respect to the mean. It is determined with the square root of the histogram variance as follows:

(9) $$\beta =\sqrt{\alpha },$$

where β is the standard deviation of the histogram. In Table 2, the histogram variance and its standard deviation are presented for plain and encrypted images. For example, the histogram variance of plain images is much higher than its corresponding encrypted images, which means the non-uniform dataset and uniform dataset, respectively. In addition, the average more-less pixel fluctuation around the mean (1024) in the R component of The Statue of Liberty 512×512 color plain image is 803 (see Figure 2b–d), whereas the corresponding encrypted component presents just 60 (see Figure 2f–g).

### 3.5. Point 5: Histogram Uniformity

An ideal uniform histogram of an encrypted image must have the same pixel frequency for all 256 intensity values. In this case, the histogram variance will be zero with a standard deviation of zero. The histogram uniformity percentage (*HUP*) is introduced to determine the intensity values falling between the acceptable range, which is defined as follows:

(10) $$HUP\left(\%\right)=\frac{1}{2.56}\sum_{i=1}^{256}\rho \left(i\right),$$

where:

(11) $$\rho \left(i\right)=\left\{\begin{array}{cc} 1 & \mathrm{if}\rho \geq \bar{x}-\beta \;\;and\;\;\rho \leq \bar{x}+\beta \\ 0 & \mathrm{otherwise} \end{array}\right.$$

where ρ is the acceptable range, $\bar{x}$ is the mean, and β is the standard deviation. For example, the *HUP* of the gray scale Lena 512×512 encrypted image and B component of The Statue of Liberty 512×512 encrypted image are 85.54% and 85.93%, respectively.

### 3.6. Point 6: Graphic Correlation

The pixel values of any plain image at any position have neighbors (horizontally, vertically, or diagonally) with pixel values that are highly similar, i.e., all plain images are strongly correlated. This natural property implies designing efficient cryptosystems to produce non-correlated encrypted images and reduce the risk of statistical attack.

The graphic correlation is a visual inspection of the pixel’s correlation of the image, where the horizontal axis represents the intensity value of the pixel and the vertical axis represents the neighbor pixel value, either horizontal, vertical, diagonal, or random. The expected graphic correlation of the plain image must present a strong pattern over a line at 45 degrees; the more close to this line, the more correlated is the tested image. In the encrypted image, the expected graphic correlation must present points over all the plane since most of the neighbors of any pixel have different intensity values with respect to that pixel.

**Figure 3:**
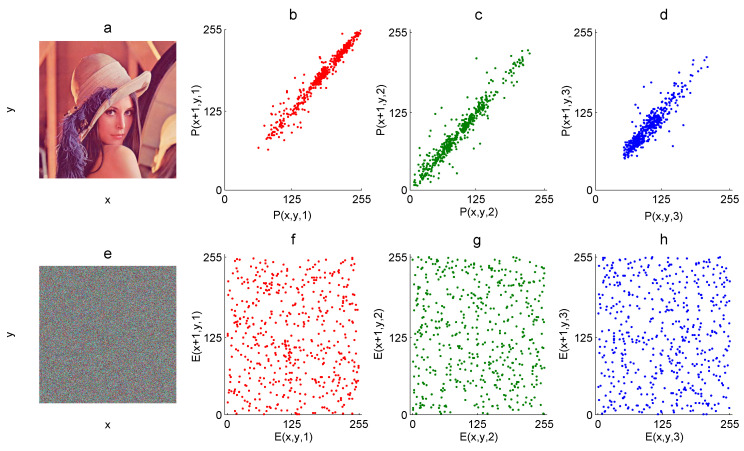
Graphic horizontal correlation: (**a**) RGB plain image *P*, (**b**) R-component of *P*, (**c**) G-component of *P*, (**d**) B-component *P*, (**e**) RGB encrypted image *E*, (**f**) R-component of *E*, (**g**) G-component of *E*, and (**h**) B-component of *E*.

In Figure 3b–d, the graphic correlation (horizontal) of 5000 random pixels of the Lena 512×512 color plain image is presented for each R, G, and B component, whereas the graphic correlation of the corresponding encrypted components is shown in Figure 3f–h.

### 3.7. Point 7: Correlation Coefficient

The Pearson correlation coefficient (*PCC*) is the metric implemented to support the visual inspection of the graphic correlation. In statistics, *PCC* measures the grade of the linear relationship between two quantitative variables by the numeric index, which varies between [−1,1]. When it is −1, there is a perfect inverse relationship, whereas when it is one, there is a perfect direct relationship. Just when *PCC* is zero, neither tested variables has a linear relationship (null correlation).

Therefore, plain images do not need to present *PCC* close to one since they are highly correlated. On the other hand, encrypted images must present *PCC* close to zero to resist statistical attacks. The *PCC* of an image can be measured as follows:

(12) $$r_{x,y}=\frac{N\sum_{i=1}^{N}\left(x_{i}y_{i}\right)-\sum_{i=1}^{N}x_{i}\sum_{i=1}^{N}y_{i}}{\sqrt{\left(N\sum_{i=1}^{N}{\left(x_{i}\right)}^{2}-{\left(\sum_{i=1}^{n}x_{i}\right)}^{2}\right)\left(N\sum_{i=1}^{N}{\left(y_{i}\right)}^{2}-{\left(\sum_{i=1}^{n}y_{i}\right)}^{2}\right)}},$$

where *x* and *y* are two variables defined by some pixel values and their neighbor pixel values, *N* is the number of pixel pairs, and $r_{xy}$ is the *PCC*. For example, 5000 random pixels of the Lena 512×512 color plain image *P* (Figure 3a) are defined as *x*, and its corresponding horizontal neighbor pixels are defined as *y*. In this case, $r_{xy}=0.9777$ for the R-component of *P*, $r_{xy}=0.9604$ for the G-component of *P*, and $r_{xy}=0.9102$ for the B-component of *P*. On the other hand, the *PCC* for the corresponding encrypted image *E* is $r_{xy}=-0.0260$ for the R-component of *E*, $r_{xy}=-0.0127$ for the G-component of *E*, and $r_{xy}=-0.0092$ for the B-component of *E*.

### 3.8. Point 8: Key Sensitivity

The encryption algorithm must be highly sensitive against the lowest change of the secret key to make all the key space efficient. The encryption and decryption process must show this important characteristic. In the encryption process, the same plain image *P* is encrypted two times with two similar secret keys ($K_{1}$ and $K_{2}$), and the corresponding cryptograms ($E_{1}$ and $E_{2}$) must be very different between them and visually unrecognizable.

This test must be performed over the smallest change at the initial condition, or the control parameter of chaotic map, or at any variable that determines the chaotic keystream used in the encryption process. For example, if the Hénon map (Equation (3)) is adopted with the IEEE 754 standard for double floating-point arithmetic, the secret key is defined with two states and two control parameters, but just one of them is lightly modified, e.g., $b_{1}=1.412345678901234$ (part of $K_{1}$) and $b_{2}=1.412345678901235$ (part of $K_{2}$).

**Figure 4:**
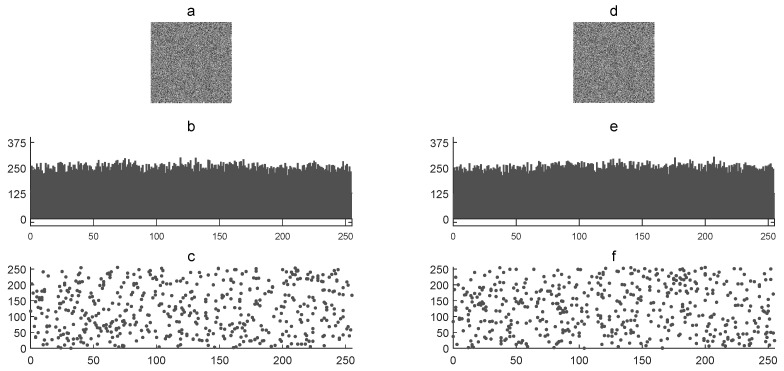
Key sensitivity analysis: (**a**) cryptogram $E_{1}$, (**b**) graphic histogram of $E_{1}$, (**c**) graphic horizontal correlation of $E_{1}$, (**d**) cryptogram $E_{2}$, (**e**) graphic histogram of $E_{2}$, and (**f**) graphic horizontal correlation of $E_{2}$.

Figure 4 shows the corresponding cryptograms ($E_{1}$ and $E_{2}$) with their graphic histograms and graphic correlations. The statistics of $E_{1}$ and $E_{2}$ are as follows, respectively: 127 and 128 for image mean $\bar{x}$, 443.50 and 414.92 for histogram variance α, 21 and 20 for histogram standard deviation β, and 0.06642 and 0.01271 for the correlation coefficient $r_{xy}$. The *NPCR* and *UACI* tests were used to determine the difference between both $E_{1}$ and $E_{2}$ cryptograms. In this case, the *NPCR* was 99.6093% and the *UACI* was 33.0435%. This visual and numerical results verified the sensitivity of secret keys in the encryption process. In addition, the statistical results were similar in both cryptograms.

In the decryption process, just the correct secret key must retrieve the plain image, whereas decryption with highly similar secret keys to the correct one must produce a similar result as encryption cryptograms both visually and statistically.

### 3.9. Point 9: Graphic Autocorrelation

**Figure 5:**
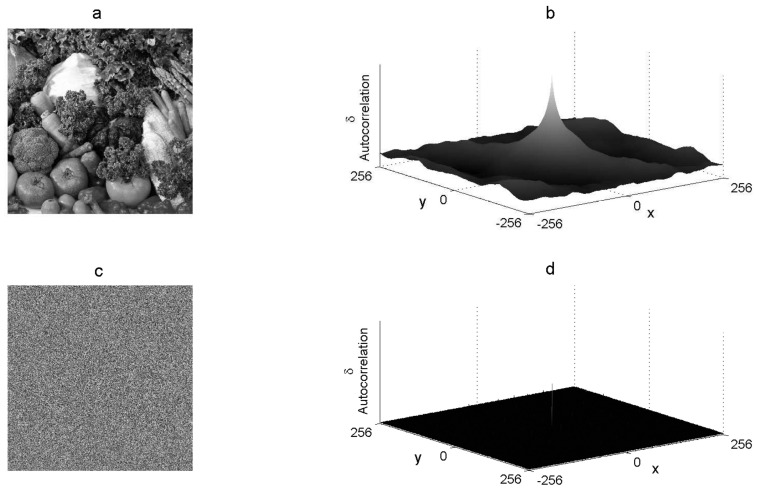
Autocorrelation graphics: (**a**) plain image *P*, (**b**) autocorrelation of *P*, (**c**) cryptogram *E*, and (**d**) autocorrelation of *E* .

The graphic autocorrelation of the 2D image compares all possible pixel pairs and expresses the probability that both pixels will have similar values as a function of the distance and direction of separation. Mathematically, the autocorrelation of an image *i* is the convolution of a function with itself, which can be calculated by:

(13) $$\delta \left(a,b\right)=\sum_{x=1}^{M}\sum_{y=1}^{N}i\left(x,y\right)*i\left(x-a,y-b\right),$$

where δ(a,b) is the autocorrelation function, i(x,y) is the image intensity at position (x,y), “∗” represents the convolution, and *a* and *b* represent the distance from the corresponding *x* and *y* position. Equation (13) is almost never used due the required high computational complexity. Autocorrelation can be calculated efficiently via fast Fourier transform by using the Wiener–Khinchin theorem described as follows:

(14) $$F\left(\delta \left(a,b\right)\right)=S\left(i\right)={\left|F\left[i\left(M,N\right)\right]\right|}^{2},$$

where S(i) is the power spectrum of the image *i* and *F* is the Fourier transform. This theorem states that the Fourier transform of the autocorrelation of image *i* is equal to the inverse Fourier transform of S(i). S(i) can be calculated by squaring the magnitude of the Fourier transform of image *i*, which is equivalent to multiplying the Fourier transform by its conjugate. Therefore, the autocorrelation of image *i* is determined by:

(15) $$\delta \left(a,b\right)=F^{-1}\left\{F\left[i\left(M,N\right)\right]\cdot \bar{F}\left[i\left(M,N\right)\right]\right\},$$

where $F^{-1}$ is the inverse Fourier transform and $\bar{F}$ is the conjugated Fourier transform. The graphic autocorrelation of the plain image must present waves and a cone in the center of the M×N graphic space, whereas an encrypted image must present a flat and uniform graphic autocorrelation. Figure 5 shows the graphic autocorrelation of both the *Vegetables*512×512 gray-scale plain image and its corresponding encrypted image.

### 3.10. Point 10: Information Entropy

Information entropy is a mathematical property that determines the randomness, unpredictability, or complexity of a message. If encryption process does not produce enough disorder at the output, the cryptosystem can be the subject of entropy attack. An encrypted image *E* with $2^{N}$ possible combinations will have maximum entropy of H(E)=N ideally. Therefore, the maximum information entropy of any gray-scale image or any RGB component image is eight. The entropy H(m) of a message *m* can be calculated as follows:

(16) $$H\left(m\right)=\sum_{i=0}^{2^{N}-1}p\left(m_{i}\right)\;log_{2}\left(1/p\left(m_{i}\right)\right),$$

where *N* is the number of bits of message *m*, $2^{N}$ are all possible values, $p(m_{i})$ represents the probability of $m_{i}$, $\log_{2}$ represents the base two logarithm, and the entropy is expressed in bits. In Table 3, the entropy results of both The Statue of Liberty 512×512 RGB plain image and its corresponding encrypted image are presented. Therefore, the entropy value close to eight in encrypted image means a highly unpredictable message; hence, the encryption algorithm can resist entropy attack.

### 3.11. Point 11: Encryption Quality

In this section, measuring techniques are used to verify the image encryption quantitatively such as mean-squared error (*MSE*), peak signal-to-noise ratio (*PSNR*), and structural similarity index (*SSIM*). The *MSE* is a parameter to measure the difference between two images, which is described as follows:

(17) $$MSE=\frac{1}{M\times N}\sum_{i=1}^{M}\sum_{j=1}^{N}{\left[P\left(i,j\right)-E\left(i,j\right)\right]}^{2},$$

where M×N is the size of the image, *P* is the plain image, and *E* is the encrypted image. The higher value of *MSE* represents better encryption quality. This MSE analysis is a useful test for a color RGB plain image and encrypted RGB color image with pixel values in the range of [0–255]. The *PSNR* (expressed in logarithmic scale and decibels) determines the ratio between the maximum possible power of a signal and the power of distorting noise that affects the quality of its representation. It is calculated by next equation:

(18) $$PSNR=20\;log_{10}\;\left(\frac{255}{\sqrt{MSE}}\right)$$

The *MSE* of efficient cryptograms is high. Therefore, the *PSNR* of encrypted images with high quality is expected to be low, less than 10 dB.

On the other hand, the *SSIM* determines the similarity between two images with a more consistent technique than *MSE*, i.e., a burred image is perceived as a bad quality image, which is consistent with *SSIM*, but not with *MSE*. *SSIM* considers the mean, standard deviation, and cross-correlation in both plain image *P* and encrypted image *E*. It is calculated with the following expression:

(19) $$SSIM=\frac{\left(2\bar{P}\bar{E}+C_{1}\right)\left(2\sigma_{PE}+C_{2}\right)}{\left({\bar{P}}^{2}+{\bar{E}}^{2}+C_{1}\right)\left(\sigma_{P}^{2}+\sigma_{E}^{2}+C_{2}\right)},$$

where:

(20a) $$\begin{array}{c} \bar{P}=\frac{1}{M\times N}\;\sum_{i=1}^{M}\sum_{j=1}^{N}P\left(i,j\right), \end{array}$$

(20b) $$\begin{array}{c} \bar{E}=\frac{1}{M\times N}\;\sum_{i=1}^{M}\sum_{j=1}^{N}E\left(i,j\right), \end{array}$$

and:

(21a) $$\begin{array}{c} \sigma_{P}=\frac{1}{M\times N}\;\sum_{i=1}^{M}\sum_{j=1}^{N}{\left[P\left(i,j\right)-\bar{P}\right]}^{2}, \end{array}$$

(21b) $$\begin{array}{c} \sigma_{E}=\frac{1}{M\times N}\;\sum_{i=1}^{M}\sum_{j=1}^{N}{\left[E\left(i,j\right)-\bar{E}\right]}^{2}, \end{array}$$

and:

(22) $$\begin{array}{c} \sigma_{PE}=\frac{1}{M\times N}\;\sum_{i=1}^{M}\sum_{j=1}^{N}\left[P\left(i,j\right)-\bar{P}\right]\;\left[E\left(i,j\right)-\bar{E}\right], \end{array}$$

where $\bar{P}$ is the mean of the plain image, $\bar{E}$ is the mean of the encrypted image, $\sigma_{P}$ is the standard deviation of the plain image, $\sigma_{E}$ is the standard deviation of encrypted image, $\sigma_{PE}$ is the cross-correlation of the plain and encrypted image, and SSIM≤1. $C_{1}={\left(K_{1}\;L\right)}^{2}$ and $C_{2}={\left(K_{2}\;L\right)}^{2}$ are used for stability, where L is the dynamic range of the pixel values (for gray-scale images, L = 255) with $K_{1}=0.01$ and $K_{2}=0.03$. The *SSIM* is one for identical images, whereas it is close to zero for images structurally different between them.

Table 4 presents the results of the encryption quality of three 512×512 color cryptograms from different plain images. The cryptogram with large values of *MSE*, *PSNR* lower than 10 dB, and *SSIM* close to zero means an efficient pseudorandom cryptogram and structurally different compared with the corresponding plain image.

### 3.12. Point 12: Decryption Error

In image chaos-based cryptosystems, the recovered image is supposed to be identical to the plain image, which is required in sensitive applications such as telemedicine, biometrics, military, and others. In some recent schemes in the literature, designers implement techniques to enhance the security such as random pixel insertion in the plain image or save some specific data in the encrypted image before sending it over the insecure channel. Therefore, the recovered image is not the same as the original plain image, and this error must be determined quantitatively. The decryption error is defined as follows:

(23) $$E\left(\%\right)=\frac{100}{M\times N}\sum_{i=1}^{M}\sum_{j=1}^{N}Q\left(i,j\right)$$

and:

(24) $$Q\left(i,j\right)=\left\{\begin{array}{cc} 0 & \mathrm{if}P(i,j)=D(i,j)\\ 1 & \mathrm{if}P(i,j)\neq D(i,j) \end{array}\right.$$

where *P* is the original plain image, *D* is the recovered or decrypted image, and E is the error in percentage. An ideal chaos-based image cryptosystem must present an error E of zero, i.e., the recovered image must be identical to the original plain image.

### 3.13. Point 13: Floating Frequency

The floating frequency analysis determines the uniformity of the encryption process for all the rows and columns of the image and the capacity of encryption to produce uniform random data for all sections of the plain image. This powerful analysis can show weak encryption sections in a cryptogram. The analysis is performed over windows of 256 elements based on the rows and columns of an image. The analysis consists of determining how many elements are different in each window. We introduce a rows’ and columns’ floating frequency of a 256×256 gray-scale image, which is determined as follows:

1. Select windows of 256 elements for each row and each column of the image.
2. Determine how many different elements are in each window.
3. Define the row floating frequency (*RFF*) and column floating frequency (*CFF*) with the corresponding frequencies of each window.
4. Determine the mean and plot for both *RFF* and *CFF*.

**Figure 6:**
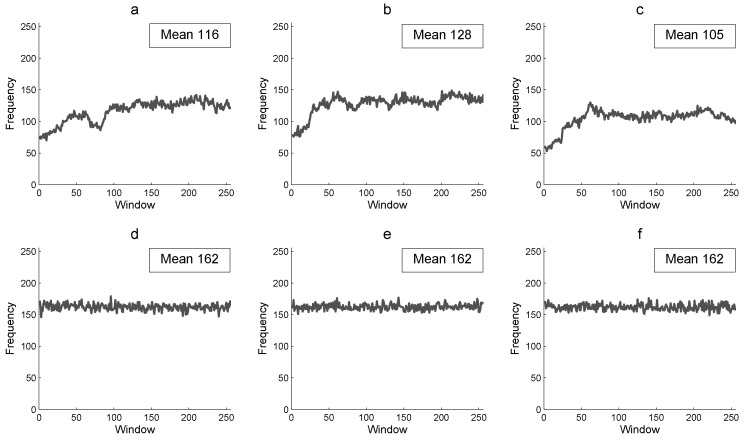
Column floating frequency and its mean: (**a**) R-component of *P*, (**b**) G-component of *P*, (**c**) B-component *P*, (**d**) R-component of *E*, (**e**) G-component of *E*, and (**f**) B-component of *E*.

Figure 6 presents the *CFF* of the Lena 256×256 color plain image *P* and of its corresponding encrypted image *E*. Figure 6a–c shows the floating frequency for each column from 1–256 of the Lena plain image, which is expected to be low and without uniformity (i.e., there are many repetitive pixels). Nevertheless, in Figure 6d–f, the floating frequency of the corresponding encrypted image shows that there are more pixels with different intensity in each column, which is desirable in the encrypted image with a mean of 162 different pixels of 256, (i.e., there are 63% different pixels for each column of 256 pixels). A greater percentage of *CFF* in the encrypted image is more efficient for the image encryption algorithm to produce a random cryptogram at column scale.

**Figure 7:**
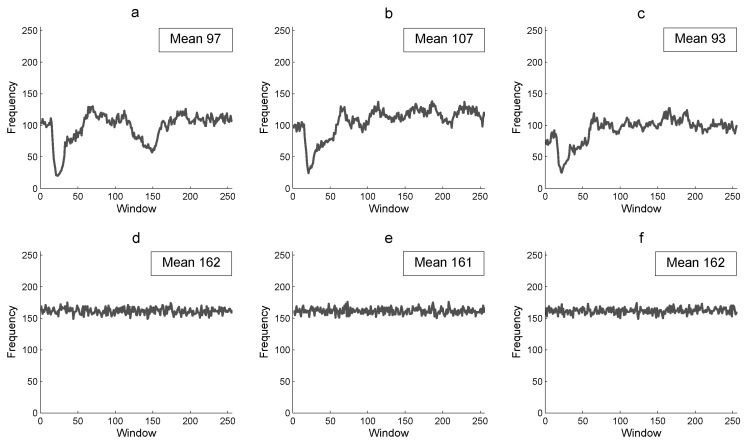
Row floating frequency and its mean: (**a**) R-component of *P*, (**b**) G-component of *P*, (**c**) B-component *P*, (**d**) R-component of *E*, (**e**) G-component of *E*, and (**f**) B-component of *E*.

Figure 7 presents the *RFF* of the Lena 256×256 color plain image *P* and of its corresponding encrypted image *E*. The results in *RFF* for the plain image are low with a mean of 97, 107, and 93 for the red, green, and blue component, respectively (Figure 7a–c). Furthermore, it shows non uniform data as expected. Figure 7c–f shows the *RFF* for the corresponding encrypted image, with a mean of 162, 161, and 162 different pixel values for each row of 256 pixels (similar results as *CFF*). A greater percentage of *RFF* in the encrypted image is more efficient for the image encryption algorithm to produce a random cryptogram at row scale.

The floating frequency is very useful to determine the capacity of the encryption algorithm to produce uniform random data for all the plain image data, i.e., the higher the floating frequency and uniformity, the higher the security and capacity of the encryption method. If floating frequency presents lower peaks in some sections, it means weak encryption in some part of the cryptogram, i.e., low capacity of the encryption process. Finally, the floating frequency test can be applied to images of 512×512 pixels by dividing it in four subsections of 256×256 pixels.

### 3.14. Point 14: Noise Robustness

The encrypted image can be alterable either by noise attack or noise jamming in the transmission channel, which can make it difficult to recover the plain image. Thus, the robustness against noise must be evaluated over chaos-based image cryptosystems. The encryption process of recent algorithms gives some advantages against noise since most of them are based on the substitution-permutation network, uniform chaos, and one-per-one pixel encryption. Considering such principles and the bulk data of images, the original plain image can still be reconstructed with high visual quality.

In noise analysis, the cryptogram is spread with salt and pepper noise with different densities by using the “*imnoise*” function of MATLAB. The percentage of affected pixels can be determined as follows:

(25) N(%)=100D,

where D∈(0,1) is the selected density of salt and pepper noise.

**Figure 8:**
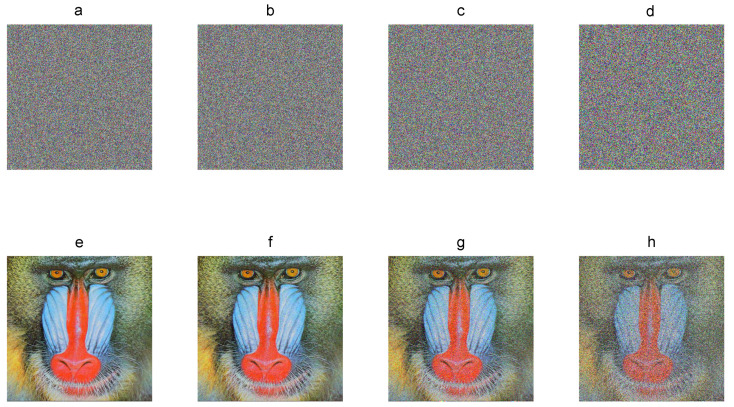
Noise robustness: (**a**–**d**) Baboon cryptograms with 1%, 5%, 20%, and 50% of noise added; (**e**–**f**) corresponding recovered images.

The decryption process is performed by using the correct secret key and cryptograms affected by noise. Figure 8 shows the cryptograms of the Baboon 512×512 color plain image with 1%, 5%, 20%, and 50% of noise and the corresponding recovered image. The original plain image can still be recovered visually with details. The decryption error E between the plain image and the recovered images is 0.95%, 4.98%, 19.92%, and 49.88% on average for the three components. Therefore, the encryption algorithm is robust against noise.

### 3.15. Point 15: Occlusion Robustness

**Figure 9:**
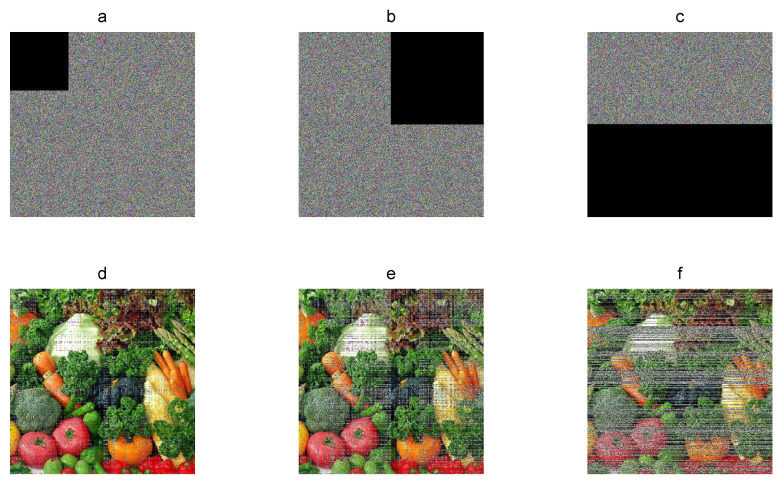
Occlusion robustness: (**a**–**d**) Vegetables cryptograms with 10%, 25%, and 50% of lost pixels; (**e**–**f**) corresponding recovered images.

The encrypted image can lose information by an occlusion attack or by transmitting over network and storage, which can make it difficult to recover the plain image. In this sense, occlusion robustness must evaluate the capacity of recovering the original image against lost data. In occlusion analysis, the encrypted image is alterable with several pixels defined as zero. For example, Figure 9 shows the recovered Vegetables 512×512 color plain images from the corresponding cryptograms with 10%, 25%, and 50% of occlusion. The original plain image can still be recovered visually with enough details. The decryption error E between the plain image and recovered images is 10.3%, 25.53%, and 50.01% on average for each component. Therefore, the encryption algorithm is robust against occlusion.

## 4. Cost and Performance

The cost and performance of image chaos-based cryptosystems must be provided in detail, since they are the next important point to evaluate after security. Cryptographic designers and cryptanalysts could decrease the performance of some highly secure cryptosystems if it is at a high cost. Therefore, the cryptosystem must provide enough security with a reasonable cost and performance. Memory requirements, computational efficiency, and speed analysis are some constraints that must be evaluated.

Most of the chaos-based image cryptography proposed in the literature is based on the symmetric-key, since it is faster than asymmetric-key cryptography. In addition, most of them are based on MATLAB (software) implementations with C code due the fast prototype design and practical environment for testing. Others are implemented with Java code for professional applications to achieve better speed in encryption. On the other hand, the cost and performance of chaos-based image cryptosystems based on hardware implementations such as in field programmable gate array (FPGAs) and application-specific integrated circuits (ASICs) are currently an interesting research area due to the wide applications, where large volumes of secure data need to be processed (such as in biometrics, telemedicine, military, pay TV, SSL accelerators, etc.), and due to the benefits in space and cost with high performance security in an embedded system. Therefore, other requirements must be determined such as power consumption or occupied logical elements (slices, LUTs, DSP blocks, etc.) for hardware applications.

### 4.1. Point 16: Memory Requirements

Chaos-based image cryptosystems require considerable memory space in both the hard drive or FLASH memory and RAM memory due to the bulk data of images and real variables for chaos implementation. When countermeasures are added to increase security and chaos uniformity, the memory requirements are more demanding.

In digital encryption/decryption algorithms, RAM is required for the secret key, plain image, encrypted image, chaotic vectors, and any other temporal variable. The encryption algorithm code size requires some mega-bytes (MB) in the hard drive on a personal computer or in FLASH memory for an embedded system. For example, the color image encryption algorithm proposed in [25] was implemented in MATLAB with 16.38 MB in the hard drive memory according to the computer’s properties. If the color plain image of a 512×512 size is encrypted, the required RAM memory is 1.68 MB approximately, according to the function “*whos*” of MATLAB (see Table 5).

### 4.2. Point 17: Algorithm Efficiency

The encryption algorithm is a step-by-step procedure written by some language, and its efficiency is highly important in terms of complexity and execution speed. The complexity is asymptotically estimated with big-*O* notation as a function that depends on the input size *n*, e.g., O(f(n)). The complexity of a series of sentences of an algorithm is of the order of the sum of the individual complexities, and some practical rules are considered, e.g.,: input-output simple sentences and *If* sentences are of order O(1), the *for* cycle is order O(1) for *k* iterations independent of the input *n* or O(n) for *n* iterations; the *for* double nested cycle is of order $O(n^{2})$ for *n* iterations for each cycle; the iterative cycles with divisive-multiplicative sentences are of order O(logn) for *n* iterations; and O(logn) in the *for* cycle with *n* iterations is of order O(nlogn).

The computational cost of most chaos-based image encryption algorithms depends on the substitution-permutation network process, where both of them can be approximated with order O(MN), where *M* and *N* are the size of the input image in pixels. This is for the two *for* cycles double nested with *M* and *N* iterations, respectively. For example, the encryption algorithm proposed in [25] can be approximated to a computational complexity of O(MN) due to the encryption process in Equation (12).

### 4.3. Point 18: Speed Analysis

The speed analysis can be presented in both encryption and decryption process in seconds, encryption throughput (ET) in bytes per seconds, and number of needed cycles to encrypt one byte (NpCB). Basic details of implementation must be provided such as the operating system, CPU with the main frequency, RAM memory size, compiler, and programming language, since speed is dependent on these characteristics.

In Table 6, the speed analysis of the encryption algorithm proposed in [25] is presented for several plain RGB color image sizes. The algorithm was implemented in MATLAB software, where a laptop with Intel(R) Core(TM) i3-5005U CPU 2 GHz, 8 GB RAM, and Windows 10 64-bit operation system were used. The speed in seconds was determined with the *tic* and *toc* MATLAB functions; the bytes per second or encryption throughput (ET) were calculated by dividing the image size (in bytes) between time encryption (in seconds); and the number of needed cycles to encrypt one byte (NpCB) was obtained by dividing the main clock frequency (in Hertz) between ET (in bytes per second). The results showed the best performance for plain images close to 200×200 pixels, since the throughput was about 10.58 MB/s and there was less NpCB in encryption. In addition, the time encryption was compared with the conventional symmetric-key AES-128 in ECB (Electronic Code Book) mode, but without considering the vector construction process (3MN)×1 (plain text/encrypted text byte-vector with a length multiple of 16) of all plain pixels before AES encryption and the recovered image construction process M×N×3 after AES decryption. The AES-128 ECB mode encryption/decryption high-level MATLAB functions were download from the MathWorks file exchange database (mathworks.com).

## 5. Algorithm and Implementation

Chaos-based image cryptosystems must meet another important aspect related to reliability, integrity, flexibility, simplicity, and ease of implementation since there are several types and formats of images and several kinds of digital platforms. The most-used bitmap image formats are BMP, GIF, JPG, TIF, and PNG. However, it is convenient to use a format that does not imply loss of information (e.g., JPG) for the encrypted image since the decryption process could not be successfully. High processing platforms such as FPGA or ASIC can be used for cryptosystem implementation at the hardware level for low-cost embedded system applications of high-definition image encryption (HD), audio encryption, and video encryption. On the other hand, high-performance microcontrollers are interesting for the software level to medium quality image encryption applications at low cost. Finally, the PC is the platform most widely implemented for image encryption algorithms based on MATLAB programming.

### 5.1. Point 19: Flexibility

Flexibility is related to the capacity of the algorithm to endure minor modifications according to the requirements. Encryption algorithms with greater flexibility are preferable since they will meet the needs of more users (assuming good overall security and performance). For example, the 3DES structure does not support any modifications (i.e., its flexibility is null), and RC6 has a variable key length until 2048 bits with a multiple of 32 bits (secret key flexibility). Examples of flexibility may include (but are not limited to) the following:

- The algorithm can be parallelized to achieve higher performance.
- The algorithm can be implemented securely and efficiently on a wide variety of platforms.
- The cryptosystem can be used with different secret key lengths.
- The cryptosystem can be used with different plain image formats.
- The algorithm can support any image size.
- The algorithm can support gray-scale images or RGB color images.
- The algorithm can be incorporated into existing protocols and applications, requiring as few changes as possible.

### 5.2. Point 20: Simplicity

The simplicity is related to the relative algorithm design simplicity to analyze and implement efficiently such as using less arithmetic operations, using simple substitution-permutation processes, including GUI (graphical user interface) with intuitive symbols and easy operation, or including GUIDE (Graphical User Interface Development Environment) by using MATLAB, among others. It is one of the most important factors in selecting cryptographic algorithms, e.g., the Rijndael algorithm was less secure than its rivals in the AES competition, but it was selected as the AES winner due to its simplicity [45]. In this sense, chaos-based image encryption algorithms must provide detail about their flexibility and simplicity.

## 6. Conclusions

Chaos-based image cryptography is currently a world-wide research topic with hundreds of papers that have been published in the last few years. The lack of security frameworks for chaos-based image cryptosystems make each author present different tests about security over the proposed algorithms. Even some algorithms have been broken in the last few years. In this sense, security guidelines are required to make chaos-based image cryptography more consistent and promising.

The suggested guideline in this paper does not intend to limit the liberty of cryptographic designers to implement new analysis, and it does not guarantee security. However, if cryptographic designers consider this framework based on 20 points, an integral analysis about the comprehensive security analysis, cost and performance, and algorithm and implementation of new chaos-based image cryptosystems can be presented more consistently in terms of security and efficiency. Consequently, future chaos-based image encryption algorithms can be compared rigorously in terms of security and efficiency capabilities. Cryptographic designers could present just the results about security in their works (e.g., in a Table) pointing out to each of the 20 points suggested in this paper without describing all details about each test.

The guideline can be adapted for other kinds of plain text in chaos-based cryptosystems, e.g., alphanumeric text, biometrics, biosignals in telemedicine, or pseudo-random number generators. Future detailed frameworks related to chaos-based image cryptanalysis could be presented to design algorithms robust against powerful attacks (for example, chosen-known plain image attack).

## Figures and Tables

**Table 1:** Security analysis presented in recent chaos-based image cryptosystems, where √ means “presented” and - means “not presented”.

Reference	[25]	[27]	[28]	[30]	[31]	[36]	[37]	[38]	[42]	[43]
Publication Year	2015	2016	2016	2017	2017	2018	2018	2018	2019	2019
Chaos										
Chaos validation	√	-	√	√	√	-	√	√	√	√
Security										
Key space	√	√	√	√	√	√	√	√	-	√
Key sensitivity	√	√	√	√	√	√	√	√	-	√
Plain text sensitivity	√	√	√	√	√	√	√	√	√	√
Graphic histogram	√	√	√	√	√	√	√	√	√	√
Histogram statistics	-	-	-	-	√	-	-	-	-	√
Histogram uniformity	-	-	-	-	-	-	-	-	-	-
Graphic correlation	√	√	√	√	√	√	-	√	√	-
Correlation coefficient	√	√	√	√	√	√	-	√	√	√
Graphic autocorrelation	-	-	-	-	-	-	-	-	-	-
Information entropy	√	√	√	√	√	√	√	√	√	√
Encryption quality	-	-	√	√	-	-	√	√	-	-
Decryption error	√	-	-	-	-	-	-	-	-	-
Floating frequency	-	-	-	-	-	-	-	-	-	-
Noise robustness	-	-	-	-	√	-	-	√	-	-
Occlusion robustness	-	-	√	√	-	-	-	√	-	-
Cost and performance										
Memory requirements	-	-	-	-	√	-	-	-	-	-
Algorithm efficiency	-	√	-	-	-	-	√	-	-	-
Speed analysis	√	√	√	√	√	√	√	√	√	-
Algorithm and implementation										
Flexibility	-	-	-	-	-	-	-	-	-	-
Simplicity	-	-	-	-	-	-	-	-	-	-

**Table 2:** Histogram statistics with the variance and standard deviation of plain and encrypted images.

Plain Image	Scale	α	β
Lena 256×256	gray	38,451	196
Lena 512×512	gray	633,397	795
	R	645331	803
The Statue of Liberty 512×512	G	1,145,519	1070
	B	1,341,306	1158
Encrypted image	Scale		
Lena 256×256	gray	414	20
Lena 512×512	gray	3340	57
	R	3620	60
The Statue of Liberty 512×512	G	3655	60
	B	3499	59

**Table 3:** Information entropy of the plain and encrypted image.

	Plain Image			Encrypted Image		
	R	G	B	R	G	B
H	7.6299	7.4161	7.2729	7.9971	7.9972	7.9972

**Table 4:** Quality metrics’ analysis.

Cryptogram	MSE			PSNR (dB)			SSIM		
	R	G	B	R	G	B	R	G	B
Vegetables	12185	10156	14574	7.27	8.06	6.49	0.0073	0.0049	0.0044
The Statue of Liberty	9452	8015	9648	8.37	9.09	8.28	0.0056	0.0058	0.0054
Lena	10677	9105	7200	7.87	8.60	9.68	0.0065	0.0049	0.0076

**Table 5:** RAM memory requirements.

Operation	RAM Memory (KB)
Input plain image	786.432
Output plain image	786.432
Input secret key	0.016
Secret key manage	0.168
Logistic Map 2	8
Logistic Map 1	40
Permutation vector	8.2
Diffusion vector	40
Other variables	12.5
Total RAM	1681.748

**Table 6:** Speed analysis for RGB image encryption and the AES-128 ECB algorithm.

RGB Color	Algorithm in [25]			AES-128 ECB		
Image Size	Time (s)	ET (MB/s)	NpCB	Time (s)	ET (KB/s)	NpCB
100×100	0.004573	6.56	304	1.704812	17.59	113700
200×200	0.011335	10.58	189	6.577855	18.24	109,649
300×300	0.026501	10.18	196	14.896973	18.12	110,375
400×400	0.054247	8.84	226	28.367620	16.92	118,203
500×500	0.079553	9.42	212	42.928550	17.47	114,481
600×600	0.116829	9.24	216	65.174625	16.57	120,700
700×700	0.157615	9.32	214	82.195552	17.88	111,856
800×800	0.202662	9.47	211	107.279753	17.89	111,794
900×900	0.253125	9.60	208	133.858570	18.15	110,192
1000×1000	0.309373	9.69	206	172.635700	17.37	115141
1080×1080	0.364824	9.59	208	190.533774	18.36	108932
